# Uterine Hemorrhage

**Published:** 1888-04

**Authors:** William Goodell

**Affiliations:** Professor of Gynecology, University Pennsylvania, Philadelphia, Pa.


### *UTERINE HEMORRHAGE.*

By WILLIAM GOODELL, M. D.,

Professor of Gynecology, University Pennsylvania, Philadelphia, Pa.

Suppose a woman, about fifty years of age, who has borne children, comes to you with the statement that at the age of forty-five the menses ceased, and that she had no discharge of blood from the vagina from that time until six months ago, when she again began to lose blood: what would you suspect? You should suspect cancer of the cervix. Why? Because as a result of her labors a laceration of the cervix has probably happened, and a carcinoma has developed in the cleft of the tear. I will venture to say that in ninety-five out of a hundred cases this diagnosis would be correct. [That was my suspicion in a case which I had placed under the care of Dr. James B. Hunter, of this city, who found only fungoid degeneration, and cured the patient by dilating and curetting the uterus. An almost identical case occurred in the practice of Dr. A. P. Dudley, who presented the material removed by the curette to the New York Pathological Society. —Ed.]

Suppose, however, that a woman, also about fifty years old, has not borne children, and that the menses have not ceased, but have continued and increased in quantity: what then should pass through your mind? You should infer that the hemorrhage is probably due to one of two factors—either to a fibroid tumor, which is the more common, or to a polypus. The fact that she has not borne children would tend to eliminate the suspicion of carcinoma; for it is exceedingly rare to find cancer of the neck of the uterus in sterile women. I have, however, seen this in two instances, one of which, however, tends to strengthen the rule. This was the case of a lady, about sixty years of age, who had a large fibroid tumor of the womb, which, in the process of enucleation, had forced open the os to the size of a silver dollar, and was protruding from it. I wrenched the tumor off and removed it. Cancer subsequently developed in the cervix, which had been injured by the long protusion of the tumor. The second exception came to my notice a few months ago. It was that of a married lady who, I am sure, has never borne a child. She had a cancer of the neck of the womb, from which she died. Carcinoma will sometimes attack the fundus of the uterus in the sterile, but this is also very rare.—*Va. Med. Monthly.*

